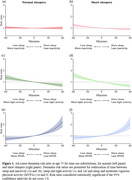# 24 hour behaviors and dementia risk

**DOI:** 10.1002/alz.091095

**Published:** 2025-01-09

**Authors:** Stephanie Yiallourou, Lachlan Cribb, Beaudan Campbell‐Brown, Christian Brakenridge, Andree‐Ann Baril, Matthew P. Pase

**Affiliations:** ^1^ Turner Institute for Brain and Mental Health, Monash University, Melbourne, VIC Australia; ^2^ Monash University, Melbourne, VIC Australia; ^3^ Swinburne University, Melbourne, VIC Australia; ^4^ Research Center of the CIUSSS‐NIM, Hôpital du Sacré‐Coeur de Montréal, Montreal, QC Canada; ^5^ Turner Institute for Brain and Mental Health & School of Psychological Sciences, Monash University, Clayton, VIC Australia

## Abstract

**Background:**

Short sleep duration, low physical activity and high sedentary time are associated with higher dementia risk. To date, previous studies have considered these behaviors in isolation, and not as inter‐related behaviors part of the 24‐h day. Compositional data analysis (CoDA) treats these behaviors as inter‐related within a constrained 24hrs. This allows for estimating the association of reallocating time from one behavior to another on health outcomes whilst adjusting for the remaining behaviors.

Using CoDA, we estimated the association of substituting sleep duration for daytime behaviors (sedentary, light and moderate‐to‐vigorous physical activity [MVPA]) on dementia risk in those with short (<6h) and normal sleep (≥6h).

**Method:**

88,654 dementia‐free participants (63 years (Q1, Q3: 56, 68); 56% female) from the UK Biobank completed 7‐day/night accelerometry (Axivity AX3). Incident all‐cause dementia was ascertained based on hospital records, death records, and primary care. CoDA isometric log‐ratios were used to examine the association between 24‐h use of time and dementia risk. Risk ratios for the associations between reallocating up to 1h sleep time to time in other activities and all‐cause dementia were estimated.

**Result:**

There were 484 incident all‐cause dementia cases over a median follow‐up of 7.2 years. Figure 1 presents the associations between discrete time‐use substitutions and dementia risk.

In persons with normal sleep duration, replacing light activity with sleep time was associated with a lowering of dementia risk (Fig. 1c). Conversely, replacing MVPA with sleep time was associated with the largest increase in dementia risk (Fig. 1e).

In persons with short sleep duration, replacing inactivity and light activity with sleep time was associated with a lowering of dementia risk (Fig. 1b‐d). In contrast to those with normal sleep duration, replacing sleep with MVPA did not lower risk of dementia in short sleepers (Fig. 1f).

**Conclusion:**

The way in which 24‐h time use, comprised of sedentary behavior, light activity, MVPA, and sleep, is balanced may have implications for dementia risk. In short sleepers, prioritising 1h of sleep over sedentary or light activity could be a beneficial for dementia prevention. These findings have the potential to inform more targeted and precise dementia risk prevention guidelines.